# Riley Oxidation of Heterocyclic Intermediates on Paths to Hydroporphyrins—A Review

**DOI:** 10.3390/molecules25081858

**Published:** 2020-04-17

**Authors:** Pengzhi Wang, Jonathan S. Lindsey

**Affiliations:** Department of Chemistry, North Carolina State University, Raleigh, NC 27695-8204, USA; pwang15@ncsu.edu

**Keywords:** aldehyde, bacteriochlorin, chlorin, dihydrodipyrrin, dipyrromethane, selenium dioxide, selenium reagent, tetrahydrodipyrrin

## Abstract

Riley oxidation of advanced heterocyclic intermediates (dihydrodipyrrins and tetrahydrodipyrrins) is pivotal in routes to synthetic hydroporphyrins including chlorins, bacteriochlorins, and model (bacterio)chlorophylls. Such macrocycles find wide use in studies ranging from energy sciences to photomedicine. The key transformation (–CH_3_ → –CHO) is often inefficient, however, thereby crimping the synthesis of hydroporphyrins. The first part of the review summarizes 12 representative conditions for Riley oxidation across diverse (non-hydrodipyrrin) substrates. An interlude summarizes the proposed mechanisms and provides context concerning the nature of various selenium species other than SeO_2_. The second part of the review comprehensively reports the conditions and results upon Riley oxidation of 45 1-methyltetrahydrodipyrrins and 1-methyldihydrodipyrrins. A comparison of the results provides insights into the tolerable structural features for Riley oxidation of hydrodipyrrins. In general, Riley oxidation of dihydrodipyrrins has a broad scope toward substituents, but proceeds in only modest yield. Too few tetrahydrodipyrrins have been examined to draw conclusions concerning scope. New reaction conditions or approaches will be required to achieve high yields for this critical transformation in the synthesis of hydroporphyrins.

## 1. Introduction

The oxidation of active methylene groups with selenium dioxide was reported in the open literature in 1932 [1]. While studies of the eponymous SeO_2_-mediated oxidation [1,2,3,4,5,6,7,8] and companion chemistry occupied Harry Lister Riley (1899–1986) [9] for a relatively short period, the 1932 publication [1] had perhaps the deepest impact among his more than four decades of publications. The role of selenium oxides as oxidants of organic compounds was apparently surmised in the late 19th century owing to the presence of the congeneric selenium oxide as an impurity in fuming sulfuric acid [10], yet it was the systematic studies of Riley and coworkers with purified SeO_2_ that captured the imagination of synthetic chemists. The impact was torrential—at least 10 overviews and summaries appeared in the ensuing dozen years [11], and a chapter in *Organic Reactions* (1948) listed >500 distinct substrates that had been subjected to reaction with SeO_2_ [10]—all of which highlight the unmet needs for synthetic transformations that were fulfilled by the advent of SeO_2_-mediated oxidation. Riley oxidation has found enduring use with application to diverse substrates, as recounted in broad-ranging reviews [10,11,12,13]. Our focus here is to comprehensively review the use of Riley oxidation in a key transformation leading to synthetic analogues of the native photosynthetic pigments. The scope of this targeted review covers the totality of all work on this topic since inception, which was in 2001.

**Chart 1:**
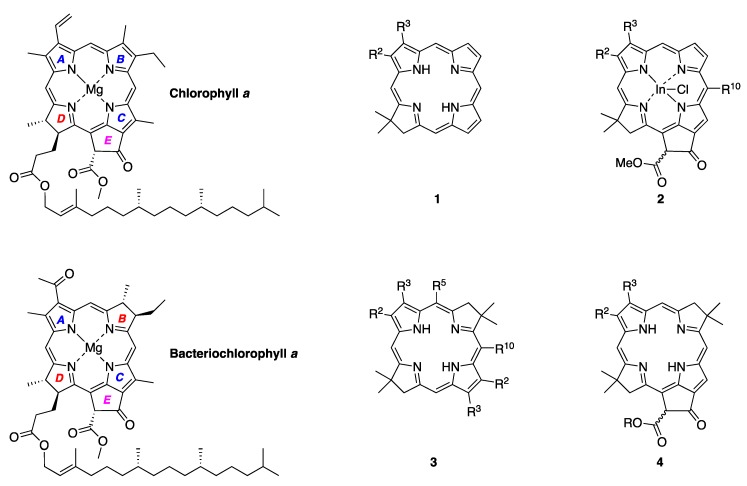
Structures of native photosynthetic pigments, synthetic chlorins (**1**, **2**), and synthetic bacteriochlorins (**3**, **4**). Each pyrroline ring (D in chlorins; B and D in bacteriochlorins) is labeled in red. The isocyclic ring (E) is labeled with magenta.

Chlorophyll *a* and bacteriochlorophyll *a*, the respective chief pigments of oxygenic and anoxygenic photosynthesis [14], are shown in Chart 1. Shown alongside the native pigments are the structures of synthetic chlorin (**1**, **2**) and bacteriochlorin (**3**, **4**) analogues. Each analogue contains a gem-dimethyl group in the pyrroline ring, and each synthesis begins with gem-dimethyl substituted precursors. Direct hydrogenation of, or cycloaddition to, a porphyrin to form the chlorin or bacteriochlorin also constitutes de novo synthesis, but the inclusion of a gem-dimethyl group via the precursors affords the following advantages: (1) achieves complete control over the location of the pyrroline ring relative to the other substituents in the macrocycle, (2) imparts resistance to adventitious oxidation that leads to the less saturated macrocycle (i.e., the chlorin or porphyrin), and (3) is compatible with formation of the isocyclic ring (ring E). On the other hand, the de novo construction of gem-dimethyl substituted hydroporphyrins entails a considerable synthetic effort [15], the simplification of which is an ongoing effort in our lab.

**Scheme 1:**
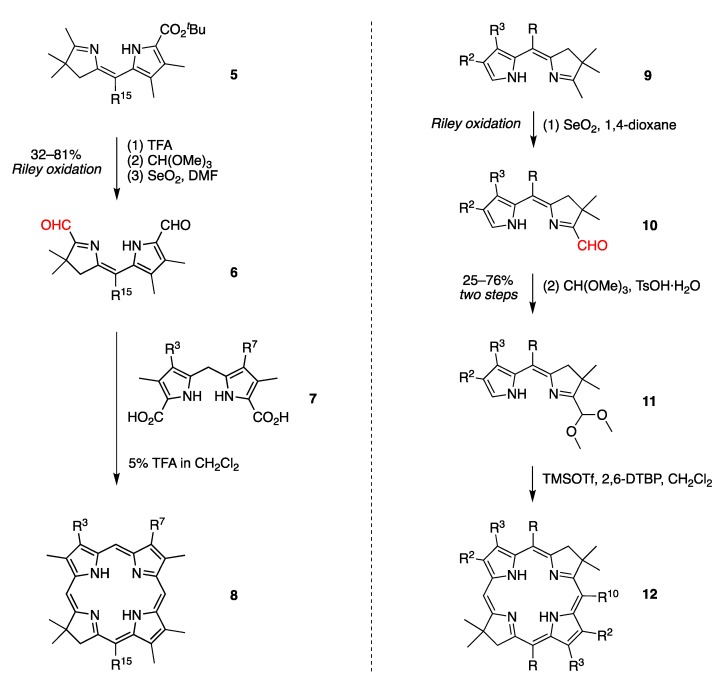
Synthetic routes to chlorins (left) and bacteriochlorins (right) via dihydrodipyrrin-carboxaldehyde precursors. TFA, trifluoroacetic acid; DMF, *N,N*-dimethylformamide; TMSOTf, trimethylsilyl trifluoromethanesulfonate; 2,6-DTBP, 2,6-di-*tert*-butylpyridine.

Two prior routes to gem-dimethyl-substituted hydroporphyrins have employed Riley oxidation as a key step in the synthesis. Jacobi and co-workers developed a route to dihydrodipyrrin-dicarboxaldehydes, wherein the aldehyde attached to the pyrrole is installed by the use of trimethyl orthoformate, and the aldehyde attached to the pyrroline is formed by Riley oxidation of a 1-methyldihydrodipyrrin (**5**) (Scheme 1, left). Acid-catalyzed reaction of a dihydrodipyrrin-dicarboxaldehyde (**6**) and a dipyrromethane-dicarboxylic acid (**7**, which undergoes didecarboxylation in situ) affords the corresponding chlorin (**8**) [16,17,18]. In our work, analogous Riley oxidation of a 1-methyldihydrodipyrrin (**9**) affords the dihydrodipyrrin-carboxaldehyde (**10**). Conversion of the latter to the corresponding dimethyl acetal (**11**) followed by head-to-tail self-condensation under acid catalysis affords the bacteriochlorin (**12**) (Scheme 1, right) [19,20]. In both synthetic routes, the gem-dimethyl group of the dihydrodipyrrin is conveyed to the pyrroline ring (ring D) of the corresponding chlorin. The pre-installation of the gem-dimethyl group in the dihydrodipyrrin enables the presence of peripheral substituents, and the positions of such substituents relative to ring D, to be set at very early stages in the synthesis. The specific location of such substituents relative to the pyrroline ring can alter the spectroscopic features, physicochemical features, and supramolecular phenomena exhibited by the resulting macrocycles.

**Scheme 2:**
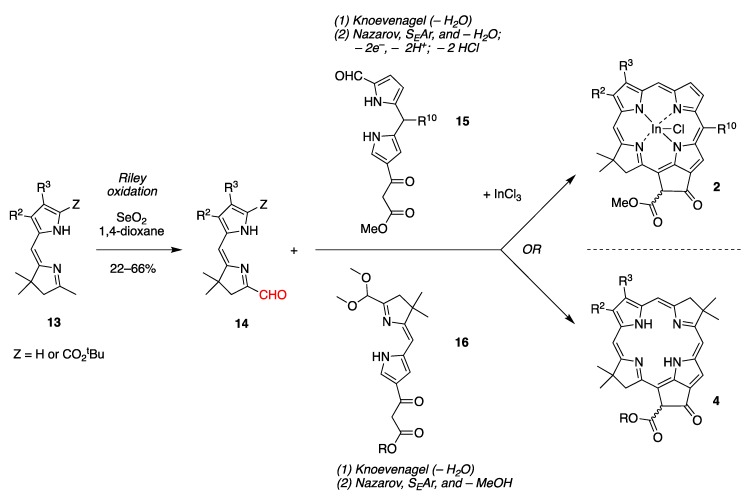
Synthetic routes to the full skeleton of chlorophylls (top) and bacteriochlorophylls (bottom) via dihydrodipyrrin-carboxaldehyde precursors.

Dihydrodipyrrin-carboxaldehydes have also been employed in more elaborate reactions leading to hydroporphyrins. The Riley oxidation of a 1-methyldihydrodipyrrin (**13**) affords the dihydrodipyrrin-carboxaldehyde (**14**), which upon Knoevenagel condensation with a β-ketoester-substituted dipyrromethane (**15**) or dihydrodipyrrin (**16**) affords the corresponding enone. Double-ring closure of each affords the model chlorophyll (**2**) [21] or bacteriochlorophyll (**4**) [22], respectively (Scheme 2). The resulting macrocycles share the full hydrocarbon skeleton with the native tetrapyrrole photosynthetic pigments, exemplified by chlorophyll *a* and bacteriochlorophyll *a*. The double-ring closure is carried out as a one-flask procedure, wherein multiple chemical transformations occur—including Nazarov cyclization, electrophilic aromatic substitution (S_E_Ar), and elimination of methanol—albeit in unknown sequence. For a definition of all abbreviations employed herein, please see the Section entitled Abbreviations. Again, the desired array of substituents in the target macrocycle is established at a very early stage of the synthesis, upon creation and/or modification of the dihydrodipyrrins for synthesis of the bacteriochlorophyll model compounds (**4**), or with the dihydrodipyrrin and dipyrromethane precursors to the chlorophyll model compounds (**2**). In both cases, the pre-arranged substituents are conveyed intact to the corresponding hydroporphyrins. Riley oxidation is essential in each of the routes (**5** → **6**, **9** → **10**, and **13** → **14**) for gaining access to the requisite dihydrodipyrrin-carboxaldehyde.

**Chart 2:**
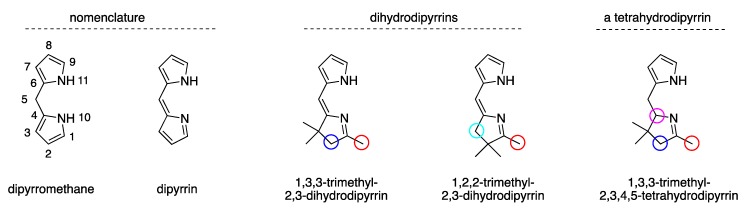
Hydrodipyrrins, nomenclature, and potential sites of allylic oxidation.

The preceding schemes illustrate the critical role of Riley oxidation in preparing a dihydrodipyrrin-carboxaldehyde for conversion to the hydroporphyrin. Yet a critical limitation is the generally low yield, often less than 50%, for Riley oxidation. Altogether, some 45 dihydrodipyrrins and tetrahydrodipyrrins have been treated with Riley oxidation as part of the synthesis of the aforementioned types of synthetic hydroporphyrins. The general structures are shown in Chart 2. As stated for the analogous tetrahydrodipyrrins [23], a dihydrodipyrrin appears quite simple, but the appearance may be deceptive because the two different heterocycles (pyrrole, pyrroline) present quite distinct reactivity as follows: (1) the pyrrole contains up to three open sites for electrophilic substitution; (2) the imine of the pyrroline, and the methylidene linkage between the pyrrole and pyrroline, are susceptible to reduction and addition; (3) the imine nitrogen can coordinate to metals; and (4) the pyrrole is a weak acid, whereas the pyrroline is a weak base.

With regards to Riley oxidation, there are multiple sites of potential reactivity. For the 1,3,3-trimethyldihydrodipyrrin, there are two aza-allylic sites: the 1-methyl group (1°, encircled in red) and the 2-methylene group (2°, encircled in blue). For the 1,2,2-trimethyldihydrodipyrrin, the 1-methyl group (1°) is an aza-allylic site, whereas the 3-methylene (2°, encircled in turquoise) presents an allylic site. For the 1,3,3-trimethyltetrahydrodipyrrin, there are three aza-allylic sites: the 1-methyl group (1°), the 2-methylene group (2°), and the 4-methine (3°, encircled in magenta); with the latter only amenable to oxidative accommodation of the hydroxy group.

The objective of this paper is to develop a comprehensive view of the outcome of Riley oxidation in these cases, particularly concerning substituent patterns and reaction conditions (solvent, temperature, additive, concentrations, time, selenium reagent). The paper is divided into two main parts. Part 1 provides a representative summary of the various conditions that have been employed, some quite sporadically, in Riley oxidations of diverse substrates (beyond dihydrodipyrrins) over the years. This section aims to cover the scope of the conditions, but is not comprehensive with regards to the scope of substrates and applications, for which other reviews are available [10,11,12,13]. Part 2 provides a review of all cases of dihydrodipyrrins subjected to Riley oxidation including structures, reaction conditions, and yields. One known dihydrodipyrrin is subjected to several reaction conditions identified in part 1. An interlude between the two parts provides an overview of mechanisms. Some insights have emerged from this comparative analysis, although a solution to the limited yields of the Riley oxidation with dihydrodipyrrins remains obscure.

## 2. Results and Discussion

### 2.1. Diverse Conditions for the Riley Oxidation

**Scheme 3:**
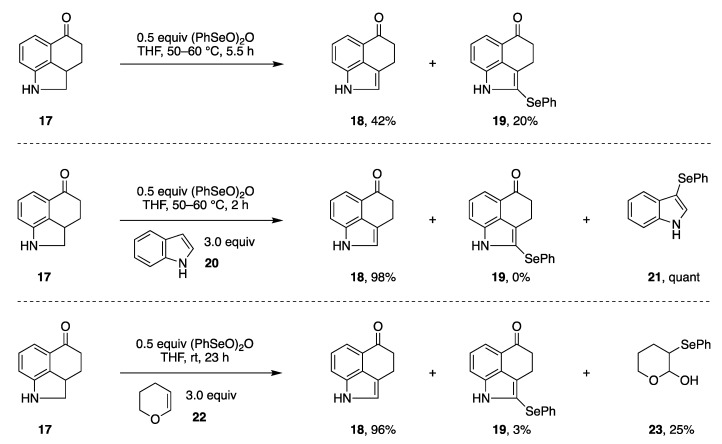
Barton’s dehydrogenation with benzeneseleninic anhydride.

The classic Riley oxidation entails the use of SeO_2_ in 1,4-dioxane (hereafter termed dioxane), either anhydrous or with a small amount of added water. A key issue here is the use of additives or other variations to the reaction conditions. While benzeneseleninic anhydride is not the same as SeO_2_, owing to the remarkable observations of Barton and coworkers, one set of examples is included. Barton and co-workers found the reaction of **17** and benzeneseleninic anhydride afforded **18** in 42% yield and the 2-selenide derivative **19** in 20% yield [24]. The addition of 3.0 equiv of indole (**20**) as a scavenger for Se(II) caused formation of **18** in almost quantitative yield, the complete absence of the unwanted derivative **19**, and the formation of the indole-trapped phenylselenide **21** (Scheme 3). Reaction with dihydropyran (**22**) as a scavenging agent afforded similar results along with formation of the dihydropyran-phenylselenide adduct (**23**). An upshot of this result is that the selenium product likely reacts with the organic product, an undesirable process that can be thwarted by the addition of a scavenging agent. The following examples focus almost exclusively on additives in reactions with SeO_2_.

**Scheme 4:**
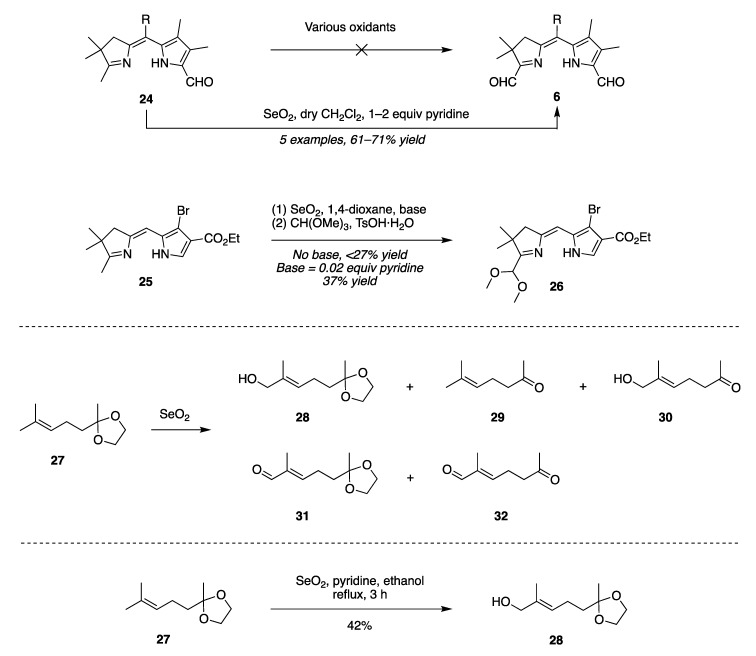
Beneficial addition of pyridine in Riley oxidations.

Pyridine is known to cause an acceleration in rate of the Riley oxidation [25]. On account of the rate-accelerating effect of pyridine and perhaps also the limited stability of dihydrodipyrrins under acidic conditions [17,26], pyridine was added to the Riley oxidation of a set of dihydrodipyrrins. Thus, dihydrodipyrrin-carboxaldehyde **24** afforded the dihydrodipyrrin-dicarboxaldehyde **6** in 61%–71% yield, and increased the yield for the conversion of **25** to **26** to 37% (Scheme 4, top). For those substrates with acid-labile groups, only a few oxidations were successful [27,28]. The reaction of **27** with SeO_2_ gave a mixture of compounds (**28**–**32**) owing to hydrolysis of the ketal (Scheme 4, middle). However, the addition of a slight stoichiometric excess of pyridine with respect to the SeO_2_ resulted in the isolation of **28** as a major product in 42% yield (Scheme 4, bottom) [29].

**Scheme 5:**
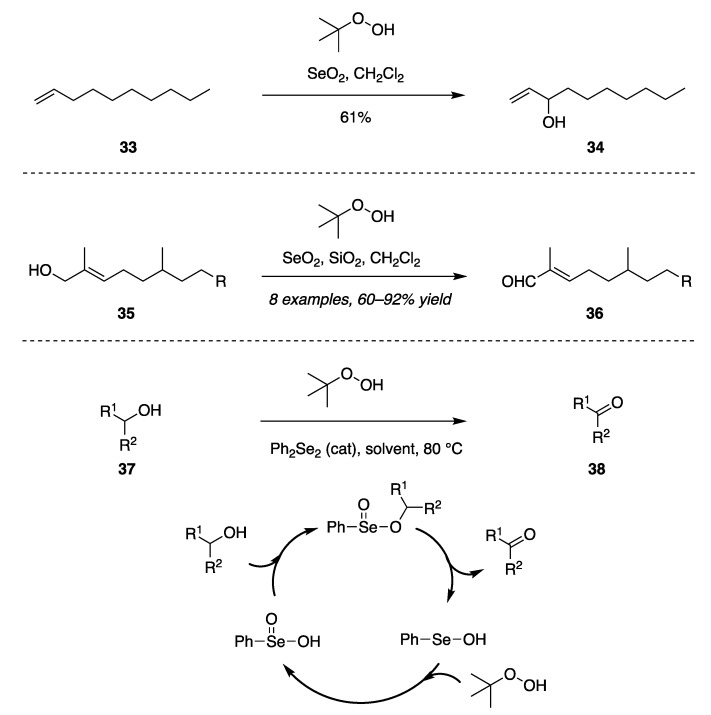
*tert*-Butyl hydroperoxide-promoted allylic oxidation of alkenes as well as oxidation of 2° alcohols.

The reaction of SeO_2_ with alkenes in the presence of hydrogen peroxide was clean for the highly reactive alkene, β-pinene, whereas less substituted alkenes reacted poorly. However, the allylic oxidation of alkenes such as **33** in the presence of 0.5 mol equiv of SeO_2_ and 2 equiv of *tert*-butyl hydroperoxide (TBHP) in CH_2_Cl_2_ afforded the corresponding allylic alcohol (**34**) in a clean and mild manner (Scheme 5, top); with other substrates, the aldehyde or ketone was similarly obtained [30]. This method also avoids many unexpected rearrangements and dehydrations that can occur under the standard conditions. In addition, SiO_2_-promoted SeO_2_ oxidation of an allylic alcohol such as **35** in the presence of TBHP gave the corresponding aldehyde **36** in 60%–92% yield (Scheme 5, middle) [31]. Recently, the catalytic oxidation with Ph_2_Se_2_ in the presence of oxygen donors such as TBHP has been used for a variety of functional groups [32,33]. The oxidation of **37** in the presence of a catalytic amount of selenium reagent and excess TBHP afforded the corresponding carbonyl compound **38** in a mild manner (Scheme 5, bottom).

**Scheme 6:**
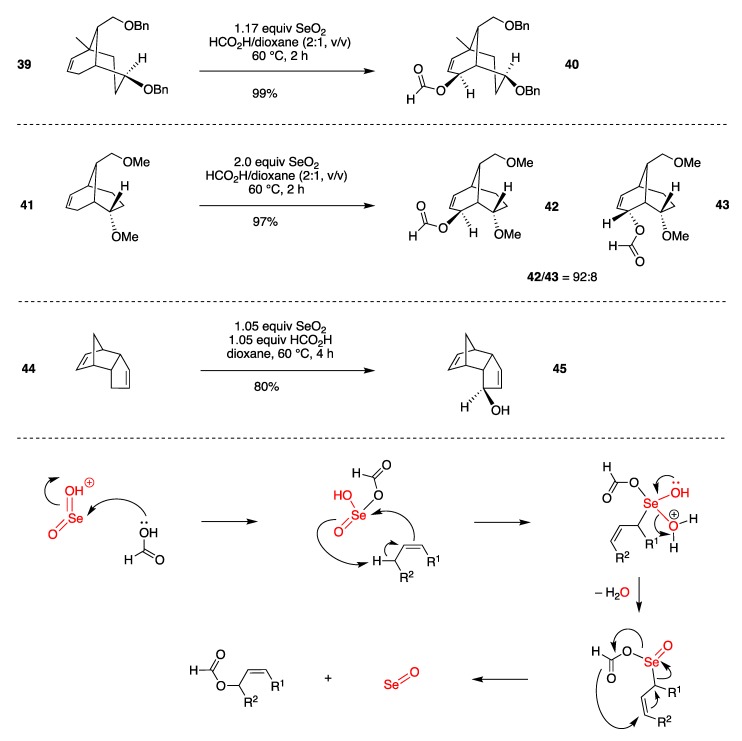
A combination of formic acid and SeO_2_ for allylic oxidation (top three panels) and a proposed mechanism (bottom panel).

The standard allylic oxidation of hindered substrates with SeO_2_ can cause undesired side reactions and leave starting material unreacted [34,35]. On the other hand, SeO_2_ oxidation of **39** in a 2:1 mixture of formic acid and dioxane afforded **40** in 99% yield, but the use of acetic acid as solvent gave a longer reaction time and lower yield of the product [34]. A combination of formic acid and SeO_2_ was found to accelerate the allylic oxidation of sterically hindered alkene **41,** affording excellent yields of the corresponding allylic formates **42** and **43** in a regioselective and stereoselective manner (Scheme 6) [35]. Application of quite similar conditions to dicyclopentadiene **44** gave the allylic alcohol **45** [35]. A proposed mechanism for oxidation with SeO_2_ in formic acid leading to the allylic formate [35] is provided in the lower panel of Scheme 6. Other discussions of mechanisms are collected in Section 2.2 (vide infra).

**Scheme 7:**
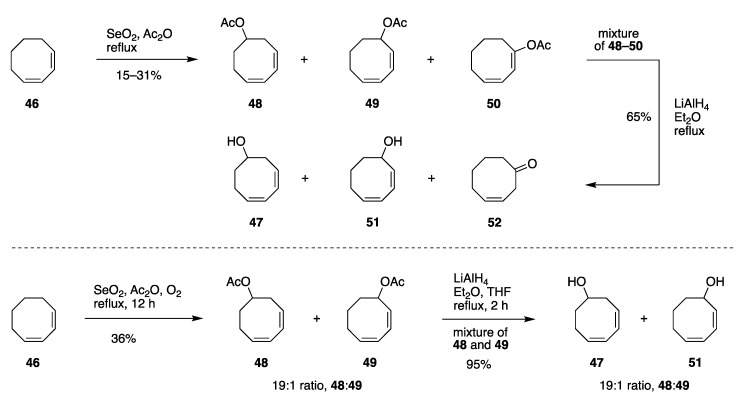
Co-oxidation of dienes with SeO_2_ and O_2_.

The conversion of cycloocta-1,3-diene (**46**) to the alcohol cycloocta-3,5-dien-1-ol (**47**) has been attempted by reduction of vinyl epoxides or by SeO_2_ oxidation of alkenes or dienes [36]. Such methods usually gave a mixture of allylic and homoallylic products and were only performed in a small scale accompanied by chromatographic purification. For example, Riley oxidation of cycloocta-1,3-diene (**46**) gave **48**, the acetate of **47**, along with two other isomeric acetates, **49** and **50**. Subsequent reduction with LiAlH_4_ gave a mixture of **47**, **51**, and **52** in 7.5:1.5:1 ratio, respectively [37]. To achieve a larger quantity of product and facilitate purification, the reaction was carried out under an atmosphere of O_2_, whereupon only two isomers (**48** and **49**) were obtained in a 19:1 ratio [36]. Herein, an SeO_2_/O_2_ combination increased the yield and selectivity for the formation of **47** (Scheme 7).

**Scheme 8:**
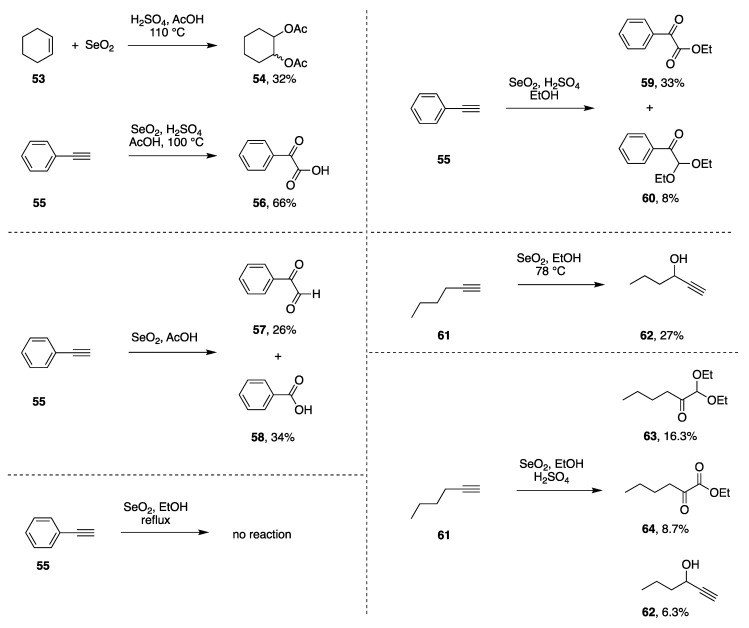
Acid-catalyzed oxidations with SeO_2_.

Selenium dioxide oxidation of alkenes in acetic acid is known to give allylic oxidation products, but in the presence of acid (H_2_SO_4_), the oxidation of cyclohexene (**53**) gave cyclohexane-1,2-diol diacetate (**54**) as the major product (32% yield) as compared with cyclohex-2-en-1-ol acetate in the absence of acid [38]. On face value, the presence of the protic catalyst promotes the formation of the direct double addition (which is still an oxidation process) versus the expected allylic substitution via the presumed organoselenium intermediate [38]. The same H_2_SO_4_-catalyzed oxidation of the acetylenic substrate **55** in acetic acid generated **56** in 66% yield. By contrast, the reaction in acetic acid gave a mixture of **57** and **58** in 26% and 34% yield, respectively; the reaction in ethanol afforded **59** and **60** in 33% and 8% yield, respectively; and in ethanol alone, no reaction occurred [39]. A remarkable change depending on reaction conditions was observed with the acetylenic substrate **61**, which has α-protons. SeO_2_ oxidation of **61** in ethanol gave the allylic product **62** in 27% yield, whereas use of a catalytic amount of H_2_SO_4_ afforded **63**, **64**, and **62** in 16.3%, 8.7%, and 6.3% yield, respectively (Scheme 8). Thus, the acid-catalyzed SeO_2_ oxidation proceeded at the triple bond rather than the α-position of the acetylenic substrate [39].

**Scheme 9:**
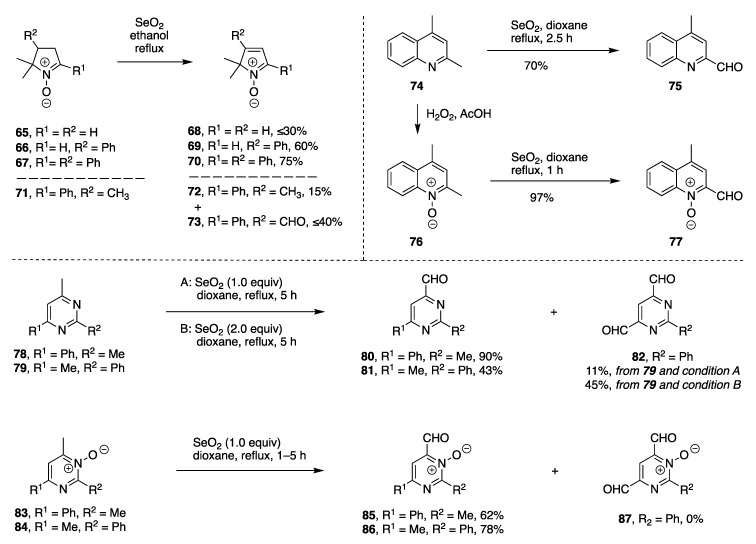
SeO_2_ oxidation of *N*-oxide substrates.

Riley oxidation has been applied to a number of heterocyclic *N*-oxide substrates (Scheme 9). In the case of pyrroline *N*-oxides lacking an α-methyl group (**65**–**67**), treatment with SeO_2_ introduced an unsaturation at the β-positions, giving the corresponding 2*H*-pyrrole (**68**–**70**). For the substrate containing a β-methyl group, but also lacking an α-methyl group (**71**), the product mixture included the corresponding β-unsaturated, 2*H*-pyrrole (**72**) and the β-unsaturated, 2*H*-pyrrole bearing a β-carboxaldehyde group (**73**) [40].

Riley oxidation of α-methyl substituted (and fully unsaturated) heterocyclic *N*-oxides generally affords good to excellent yields of the corresponding aldehyde [41,42]. Good comparisons are provided within families of methyl-substituted quinolines and of methyl-substituted pyrimidines [41]. Thus, the oxidation of a free base dimethylquinoline (**74**, lacking the *N*-oxide) gave the corresponding aldehyde **75** in 70% yield, to be compared with 97% for conversion of the analogous quinoline *N*-oxide **76** to the aldehyde **77**. The oxidation of pyrimidines **78** and **79** gave the corresponding aldehydes **80** and **81** in 90% and 43% yield, respectively, and **79** also gave the dialdehyde product **82**. The *N*-oxide substrates **83** and **84** did not give a substantially higher yield of the corresponding aldehydes **85** and **86** upon Riley oxidation versus that of **78** and **79**; however, for the *N*-oxide, a monoaldehyde was formed to the exclusion of any dialdehyde **87** (Scheme 9). In this case, the improved reaction selectivity and yield of the monoaldehyde with *N*-oxide substrates must stem in part, if not wholly, from the greater ease of formation of the corresponding enamine, although the greater stability of the heterocycle-*N*-oxide versus the parent heterocycle toward indiscriminate oxidation (e.g., removal of an electron from the heterocyclic nucleus) as opposed to site-specific SeO_2_-mediated oxidation cannot be discounted.

**Scheme 10:**
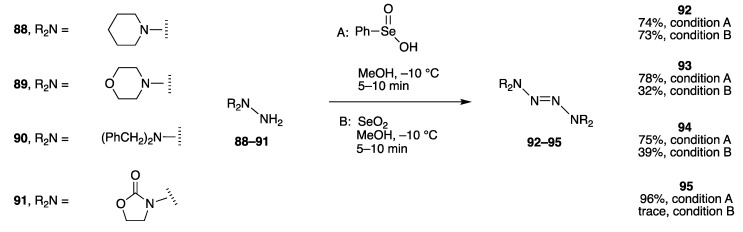
Benzeneseleninic acid versus SeO_2_ for oxidation of 1,1-disubstituted hydrazines.

Some seleninic acid derivatives can be used in conjunction with SeO_2_ to improve the reaction. One example shown in Scheme 10 illustrates the efficient oxidation provided by benzeneseleninic acid and its anhydride of various hydrazines (**88**–**90**) and hydrazo (**91**) derivatives. The hydrazines afforded the azo products (**92**–**94**) in yields that varied by <2-fold. On the other hand, an extreme case of reagent distinction is provided by **91**, where the yield of azo product **95** was 96% with benzeneseleninic acid compared with a trace amount of product upon use of the classic SeO_2_ conditions [43].

**Scheme 11:**
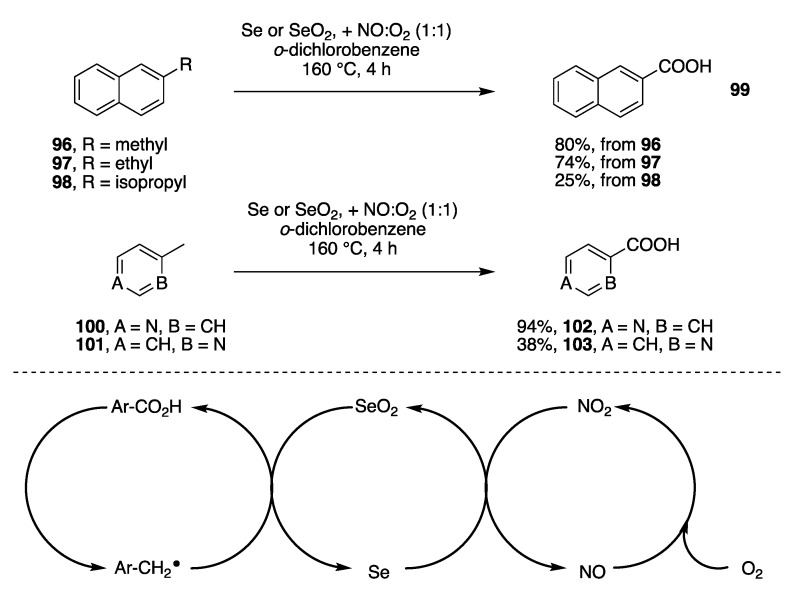
Benzylic oxidation in the presence of Se or SeO_2_/O_2_/NO.

The Riley oxidation often presents challenges in purification owing to the presence of a stoichiometric if not excess quantity of SeO_2_. The reaction can be carried out catalytically by the addition of stoichiometric oxidants such as TBHP or O_2_, both of which are desirable from economic, health, and environmental standpoints, as well as easing the challenges of purification. A method for benzylic oxidation that employed O_2_ in excess in conjunction with catalytic amounts of nitric oxide and Se or SeO_2_ was applied to a series of 2-alkylnaphthalenes (**96**–**98**), affording the corresponding naphthalene-2-carboxylic acid (**99**) (Scheme 11, top) [44]. The same reaction with picolines **100** and **101** afforded the isonicotinic acid (**102**) and picolinic acid (**103**), respectively [44]. In both reaction sets, the yields spanned a considerable range, from 25% to 80% for **99**, and 38% and 94% for **103** and **102**, respectively. The mixture of nitric oxide and O_2_ recycles reduced selenium (elemental selenium and/or other partially reduced selenium species), and thereby maintains the selenium in the catalytically active, oxidized form (Scheme 11, bottom; shown for elemental Se).

**Scheme 12:**
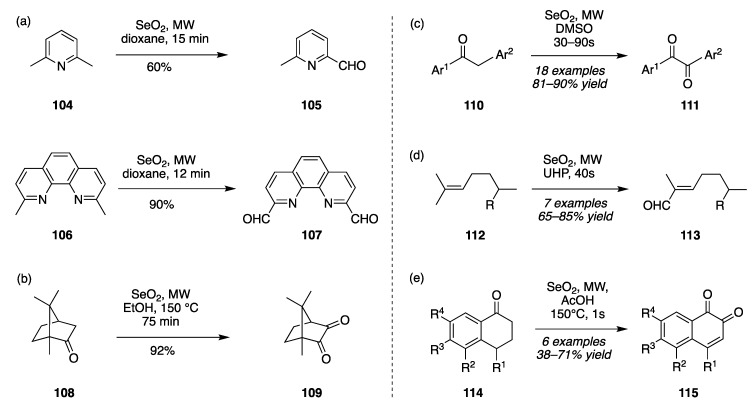
Microwave-assisted SeO_2_ oxidation. UHP, urea-hydrogen peroxide; MW, microwave.

Microwave-assisted SeO_2_ oxidation of some aromatic substrates was found to improve reaction rates and form a cleaner product; the shorter reaction times often enabled the use of a lesser excess of SeO_2_, thereby facilitating the workup. A first set of examples includes conversion of 2,6-lutidine (**104**) and neocuproine (**106**) to the respective products **105** and **107** (Scheme 12, a) [45]. The oxidation of camphor (**108**) and derivatives by SeO_2_ is often sluggish (15 h–14 days), whereas the microwave-assisted process shortened the reaction time to 75 min and produced the corresponding product (**109**) in good yield (Scheme 12, b) [46]. The microwave-assisted SeO_2_ oxidation of 1,2-diarylethanones (**110**, representing 18 compounds) to form the diones **111** also shortened the reaction time from 8 h to 30–90 s (Scheme 12, c) [47]. The nature of the aryl groups in **110** included considerable diversity in Ar^1^ (= X-phenyl, where X includes –H, –F, –Cl, –Br, –CH_3_, –OCH_3_, –SCH_3_; 2- and 4-positions only) and also Ar^1^ = thiophen-2-yl, but was more limited for Ar^2^ (= X-phenyl, where X includes –H, –NO_2_, –Cl, and –OCH_3_; 2- and 4-positions only). In addition, the inclusion of urea-hydrogen peroxide (UHP) and application of microwave-assisted SeO_2_ oxidation of alkenes (**112**) shortened the reaction time to 40 s and increased the yield of the corresponding α,β-unsaturated aldehydes (**113**) (Scheme 12, d) [48]. The substituents accommodated in the R group of **112** include an ethyl group terminated with –OH, –OAc, or –Br; an oxo group; an ethylidene acetal; a 2-acetoxyethylidene group; and an acetoxymethyl-substituted oxiranyl group [48]. More recently, closed-vessel microwave (CVMW) irradiation accelerated the SeO_2_ oxidation of 1-tetralones (**114**) to 1,2-naphthoquinones (**115**) to the remarkably brief period of 1 s, compared with 4–7 h upon refluxing in acetic acid (Scheme 12, e) [49].

In summary, the conditions explored over the years beyond the classic Riley oxidation (SeO_2_ in dioxane) include the following:

- Selenium reagent benzeneseleninic acid in methanol.
- Selenium reagent benzeneseleninic anhydride with indole or dihydropyran as a scavenger.
- SeO_2_ in dioxane with an added base such as pyridine.
- SeO_2_ in CH_2_Cl_2_ with the oxygen donor TBHP.
- SeO_2_ in CH_2_Cl_2_ with the oxygen donor TBHP and SiO_2_.
- Selenium reagent Ph_2_Se_2_ in CH_2_Cl_2_ with the oxygen donor TBHP.
- SeO_2_ in a mixture of formic acid and dioxane.
- SeO_2_ in acetic anhydride under an atmosphere of O_2_.
- SeO_2_ in acetic acid or ethanol with H_2_SO_4_ as an acid catalyst.
- Se or SeO_2_ in *o*-dichlorobenzene purged with a mixture of nitric oxide and O_2_.
- Microwave-assisted SeO_2_ oxidation in dioxane.

### 2.2. Mechanistic Considerations

The mechanistic course of the Riley oxidation has been the subject of investigation for more than three-quarters of a century [10]. Prior to delving into mechanism, perspective may be provided by the consideration of an experimental procedure reported in 1935 by H. A. Riley and A. R. Gray in *Organic Syntheses* [50,51]. (Note: the authors of this review believe that the cited contribution likely is that of H. L. Riley with typographical replacement of A for L. While the aforementioned publications in *Organic Syntheses* list no information concerning institutional affiliation, our supposition is posited on (i) familiarity with typewriters and typed print from that era, wherein A could be easily misread for L; (ii) the topic; (iii) publication in 1935, so soon after the original discovery; and (iv) the absence of any other publications concerning chemistry by an H. A. Riley in the period 1920–1950 as concluded from a search in Web of Science across all databases; moreover, there are only six publications to A. R. Gray, suggesting the latter likely was a research group member with H. L. Riley.) The balanced reaction for Riley oxidation of acetophenone to form phenylglyoxal is provided below (Equation (1)), where the inorganic products are elemental selenium and water. The reaction was carried out in dioxane containing 1.2 molar equivalents of H_2_O relative to SeO_2_.

C_6_H_5_COCH_3_ + SeO_2_ → C_6_H_5_COCHO + Se + H_2_O (1)

**Figure 1:**
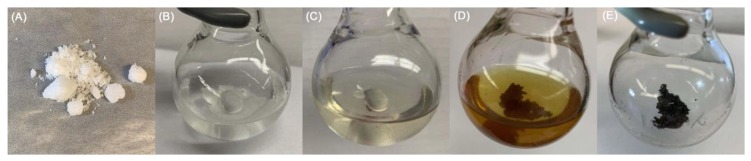
Photographs pertaining to the Riley oxidation of acetophenone. (**A**) SeO_2_ (1.1 g); (**B**) 1.1 g of SeO_2_ in 10 mL of dioxane containing 0.20 g of H_2_O; (**C**) the reaction mixture after heating at 55 °C for ~2 h and addition of 0.1 g of H_2_O to “dissolve” the SeO_2_; (**D**) the reaction mixture after addition of 1.2 g of acetophenone (1.0 M) and refluxing for 4 h; (**E**) the solid residue of putative selenium after decanting the supernatant of the crude reaction mixture.

Riley and Gray make several comments [50,51] that are germane to this discussion. First, that “commercial selenious acid (129 g, 1 mol) may be used in place of the mixture of selenium dioxide and water”. Second, referring to the mixture of SeO_2_ and water in dioxane, “the mixture is heated to 50–55 °C and stirred until the solid has gone into solution”, whereupon acetophenone is then added. Third, at the end of the reaction, that “the hot solution is decanted from the precipitated selenium”. The first and second statements highlight the question concerning the nature of the oxidizing species, while the third points to the heterogeneity of the process regardless of whether there is a homogeneous solution at the outset. The oxidation of acetophenone to form phenylglyoxal (Figure 1) was carried out by one of us following the protocol of Riley and Gray (except at 1/100th scale and at 1.0 rather than 1.7 M). The presence of water is required to achieve a homogeneous solution with SeO_2_ in dioxane. A black precipitate forms early in the reaction upon refluxing in the presence of acetophenone. Note that acetophenone is colorless, whereas phenylglyoxal is light yellow. The same reaction entirely at room temperature did not yield an initial homogeneous solution, but did afford a red precipitate.

**Scheme 13:**
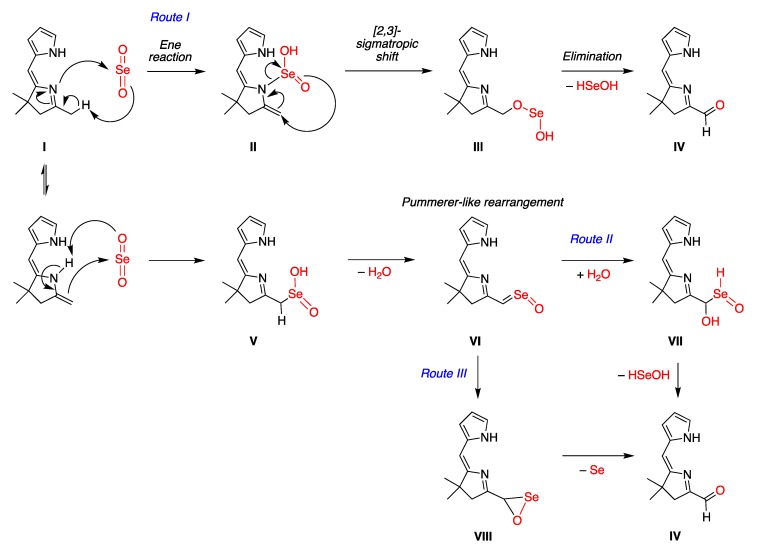
Mechanisms proposed over the years for Riley oxidation (displayed for a 1-methyldihydrodipyrrin).

At least three pathways for the mechanism of Riley oxidation have been proposed over the years [52,53,54,55,56,57,58,59]. Three composite pathways are shown in Scheme 13. The pathways, which have not been discussed previously for reactions of hydrodipyrrins, are shown here in the context of dihydrodipyrrin-carboxaldehyde formation from the corresponding 1-methyldihydrodipyrrin **I**. A distinct pathway proposed for the oxidation in the presence of formic acid, leading to allylic formates [35], is shown in Scheme 6.

- Route I entails an ene reaction of **I** and SeO_2_ to give intermediate **II**. The subsequent intramolecular [2,3]-sigmatropic shift of **II** gives **III**, which, upon elimination, generates **IV**. The latter could proceed via reductive elimination or by hydrolysis followed by redox transformations.
- Route II begins with imine–enamine tautomerization of **I**. The enamine of **I** reacts with SeO_2_ to generate intermediate **V**, and then Pummerer-like rearrangement via intermediate **VI** yields **VII**. Subsequent elimination affords **IV**.
- Route III has an alternate endgame, wherein the Pummerer-like intermediate **VI** cyclizes to give the selaoxirane-containing **VIII**, which, upon loss of Se, gives **IV**.

The mechanisms displayed are formal and encapsulate key pathways drawn from multiple reports in the literature. The term formal here refers to electron counting, use of SeO_2_ alone as an intact species, and production of minimal selenium byproducts. Several points [60] germane to any contemplation of mechanism are as follows: (1) SeO_2_ in the solid state is a polymer; (2) SeO_2_ hydrates reversibly to give selenous acid (H_2_SeO_3_; known as selenious acid in the older literature); (3) SeO_2_ is insoluble in many organic solvents, whereas selenous acid is more soluble; (4) water is often added to the reaction mixture [1] to “dissolve” SeO_2_; (5) the common oxidation states of selenium are −2, 0, +2, +4, and +6; and (6) elemental selenium forms multiple allotropes (red, black, grey) including ring species (e.g., *cyclo*-Se_8_) akin to those for elemental sulfur.

**Scheme 14:**
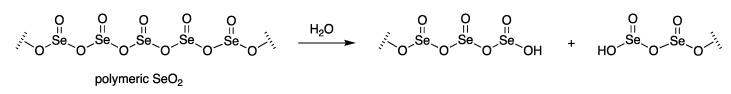
Polymeric SeO_2_, and the formation of oligomers upon partial aqueous hydrolysis.

The polymeric selenium dioxide (SeO_2_)_n_ is shown in Scheme 14. The structure resembles a polycarbonate with replacement of carbon by selenium. Treatment with a limiting amount of water produces oligomeric species containing selenous acid-like end groups. Hydrolysis with a stoichiometric quantity of water would afford a quantitative yield of selenous acid. Selenous acid is a reasonably strong acid, with *p*K_a_ ~2.5 (H_2_SeO_3_ → HSeO_3_^−^ + H^+^) [60]. One expects the terminal selenous-acid like end-groups of the oligomers derived by hydrolysis of polymeric SeO_2_ to have similar acidity. If so, one rationale for the addition of pyridine or another base to the reaction mixture in Riley oxidation may be to neutralize the resulting Bronsted acid.

A minimum conclusion from the above points is that the nature of the reacting selenium oxide species, invariably displayed as the three-atom entity SeO_2_ in textbook presentations, may include more complex substances. For the mechanisms shown in Scheme 13, the eliminated selenium byproducts (Se or HSeOH)—often not displayed explicitly in reports wherein mechanisms are proffered—are formal entities, and in fact, relatively little data are typically available concerning the composition of the selenium products, which is understandable given the focus by synthetic chemists on the organic product of the reaction. In his first paper, Riley described the appearance, recovery, and regeneration of the precipitated selenium species [1], and a selenium-containing insoluble orange, red, or black film inside the flask upon quenching the Riley oxidation (by addition of water or base) has been noted by many, including Jacobi and coworkers, who performed the first Riley oxidations of hydrodipyrrins [17], the focus of the present review. Early reviews described reports of complexes of organic substrates and selenium species in the precipitates, as well as controversies about mechanism [10]. The complexity of oxoselenium chemistry precludes simple correlation of selenium products with proposed organic mechanisms; in this regard, a formal species such as HSeOH could in principle undergo reaction, disproportionation, or combination with SeO_2_ or other species to form polyselenides and/or other products (as one hypothetical example, HSeOH + SeO_2_ → H_2_SeO_3_ + Se); similar reactions may occur with SeO_2_ (or oligomeric species thereof) with O–Se–OH moieties attached to an intermediate (e.g., **III** or **V**). If multiple selenium oxide species are present, one or more of a multiplicity of pathways may prevail, particularly under various conditions—as one explicit example, acidic conditions that cause protonation of a heterocyclic nitrogen atom may shift a reaction toward one pathway that is not a significant conduit for reactant to product under neutral conditions, and vice versa. Thus, the three mechanisms displayed in Scheme 13 are shown here for completeness as well as consideration of the results obtained upon Riley oxidation of diverse substrates.

### 2.3. Riley Oxidation of Diverse Hydrodipyrrins

**Scheme 15:**
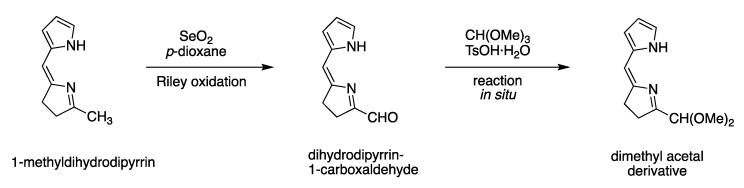
Conversion in situ of dihydrodipyrrin-1-carboxaldehyde to the corresponding dimethyl acetal, illustrated for the unsubstituted substrates.

The following tables contain examples of reactions of 45 distinct substrates including tetrahydrodipyrrins (entries 1–3) and dihydrodipyrrins (entries 4–45). A key organizational feature is that entries 1–17 contain hydrodipyrrins with gem-dialkyl groups at the 3-position, whereas entries 18–45 pertain to hydrodipyrrins with gem-dialkyl (or diphenyl) groups at the 2-position. For many cases, the yields range from 20%–60%, although some cases are reported to fail completely, while others give yields exceeding 60%. In most cases, the aldehyde is isolated, whereas in some cases, the aldehyde is converted in situ to the dimethyl acetal (Scheme 15). Isolation of the Riley oxidation product as the dimethyl acetal is provided in entries 7, 8, 18, 19, 21–28, 30–32, and 34. In rare instances, the Riley oxidation product was directly subjected to bacteriochlorin-forming conditions (entries 10 and 44), in which case the yield of the oxidation alone is obscured as one step in a three-step process (Riley oxidation, dimethyl acetal formation, and self-condensation to form the bacteriochlorin).

A few compounds listed as entries in Table 1 have been described in the preceding text. The dihydrodipyrrins presented in five entries (39b, 40b, 41b, 42, and 43) correspond to structure **24** in Scheme 4. Similarly, compound **25** (Scheme 4) is shown in entry 25. The dihydrodipyrrin shown in entry 16a was examined under various Riley oxidation conditions, and the results are presented here (entries 16b–e). The dihydrodipyrrin shown in entry 17 (compound **116**) was synthesized for this review. The synthesis procedure and characterization data for **116** are provided in Appendix A.

Examination of Table 1 for Riley oxidation of diverse 1-methyldihydrodipyrrins and several 1-methyltetrahydrodipyrrins leads to a number of insights. Concerning solvent, dioxane and DMF were usually employed. In some cases, pyridine was also added as a base. Jacobi and coworkers reported that the use of sublimed SeO_2_ and a “wet” solvent did not substantially increase the yield, and even a trace amount of water accelerated decomposition [17]. Concerning temperature, most reactions were carried out at room temperature. On the other hand, the Jacobi and Lash groups often performed the oxidation for several hours at room temperature, and then at 80 °C for 15 min (entries 35, 36a, 37, 38, 39b, 40b, 41b, 42, and 43). No examples are known of reactions at a lower temperature (<0 °C). The most significant insights concern structural effects. The interpretations drawn from the results in the table must be provisional in many cases given that, often, only single instances are available for comparison, and the synthetic work was carried out at a range of scales with various purification methods by different experimentalists. With those caveats, the insights to date include the following:

- A pyrroline *N*-oxide provides superior results (entry 1 versus 2, 79% versus 0%).
- Two 1-methyltetrahydrodipyrrin-*N*-oxides (entries 1 and 3) could be converted to the corresponding aldehyde, but neither product was subsequently converted to a hydroporphyrin. Methods for *N*-deoxygenation will likely be required to do so.
- β-Alkyl versus β-aryl groups afford comparable results (entry 6 versus 4, ~40%; and 26 versus 27, 31%).
- An aza-spirohexyl group in lieu of a gem-dimethyl has no adverse effect (entries 7,8 versus 4; ~40% for both; the former are dimethyl acetals).
- β,β-Dialkyl or β,β-annulated arenes afford comparable results (entries 9, 11 and 12; ~60%).
- A *tert*-butyl ester and ethyl ester at the 9-position afford comparable results (entries 9 and 12; ~60%).
- A pre-existing aldehyde group on the pyrrole unit survives intact and causes no adverse effect (entries 13 and 39–43; all yields >60%).
- The presence of a single aryl-substituted pyrrole gives yields of 22%–57% (entries 14–16).
- A lone *p*-bromophenyl group on the pyrrole unit affords acceptable results (entry 16, 38%), as does a *p*-iodophenyl group (entry 15, 57%), whereas a lone bromine atom on the pyrrole unit results in failure (entry 17, 0%) unless the pyrrole is stabilized with an ester substituent (entry 25, 37%; a dimethyl acetal) or a pyrrole *N*-tosyl group (entry 3, 43%; also a pyrroline *N*-oxide). Halopyrroles lacking stabilizing (e.g., electron-withdrawing) substituents are known to be unstable [66].
- A *meso*-alkyl or *meso*-aryl group affords comparable results (entries 19 and 20; 63% and 65%; the former is a dimethyl acetal).
- A *meso*-alkyl group has no apparent adverse effect (entries 42 and 43 versus 39a; >60%).
- The position of the gem-dimethyl group at the 2,2- versus 3,3-site has relatively little adverse effect (entry 23 versus 4; 25% for the dimethyl acetal versus 32 or 40% for the aldehyde).
- The presence of larger 2,2-dialkyl groups is satisfactory (entries 29 and 30; yields >50%, the latter is a dimethyl acetal).
- In one case, a *Z*-isomer gives the *Z*-isomer (entry 29, 57%), whereas the *E*-isomer gave a ~4:1 mixture of the *Z-* and *E*-products (entry 30; 51% and 12%; both dimethyl acetals).
- In another case, the *Z-* and *E*-isomers individually each give a mixture of the *Z* and *E* products (entries 31 and 32; total yields >55%; all dimethyl acetals). In this and the preceding example, the 2-position substituents are bulky (alkyl or phenyl) groups.
- 2,2-Diphenyl substituents afford both the *Z*- and *E*-isomers in comparable quantities and nearly twice the yield of the 2,2-dimethyl unit (entry 31 versus 18; 55% total versus 30%).
- In yet another case, the *Z-*isomer gives a 5:1 mixture of the *Z* and *E* products (entry 40a).
- The remarkably high yields of 99% (entries 13 and 40a) are hard to reconcile with yields of ~60% for nearly identical substrates (entries 12 and 39).
- The presence of a single ester substituent on the pyrrole unit affords good yield, whereas the fully unsubstituted pyrrole does not afford product (entry 34 versus 45; 47% versus 0%; the former is a dimethyl acetal).
- The presence of an unsubstituted pyrrole affords products that are not stable or are formed in low yield (entries 44, 45; 5.8% for the bacteriochlorin product of the former, 0% for the latter).

## 3. Outlook

Selenium—discovered in 1817 (by Berzelius and Gahn) and named after the moon (Gk, *selene*) [60]—has found myriad use in the materials sciences (e.g., photoconductors, semiconductors) and in organic chemistry, all with no sign of eclipse through >200 years of study. In organic chemistry, selenium finds its most widespread use in the SeO_2_-mediated conversion of a methyl group to the corresponding aldehyde group, which originated with the pioneering work of Harry Lister Riley in the early 1930s. In tetrapyrrole chemistry, Riley oxidation provides an essential transformation of 1-methylhydrodipyrrins to the corresponding hydrodipyrrin-carboxaldehyde or dimethyl acetal thereof. The comprehensive review here shows that the Riley oxidation has considerable tolerance for substituents in the pyrrole and pyrroline ring of the dihydrodipyrrin, although in general, the yields rarely exceed 70%, with yields of 30%–40% often more typical. Hardly any data are available concerning the nature of the side reactions, and byproducts formed, that account for the low yields. In context, most substrates examined to date for Riley oxidations have contained ketones or alkenes rather than heterocycles or imines, as described herein. Particular structural limitations that cause failure of the Riley oxidation with hydrodipyrrins include the absence of any substituents in the pyrrole nucleus, or the presence of a lone bromine atom. On the other hand, a single ester or even aryl substituent in the pyrrole nucleus suffices to give a successful oxidation. Such structural limitations impact the scope of available hydroporphyrins. The survey in part I here of a broad range of substrates reveals diverse conditions beyond those considered for the classic Riley oxidation, namely SeO_2_ in 1,4-dioxane. Many observations that bear on mechanism have been reported over the years, but fundamental mechanistic studies (which largely petered out in the latter part of the 20th century) may warrant renewed investigation, particularly in the context of the available diversity of reaction conditions. Most such reaction conditions have not been applied to the hydrodipyrrins, which may present new synthetic opportunities as the Riley oxidation nears its century mark.

## Figures and Tables

**Table 1:** Summary of Riley oxidation of hydrodipyrrins *^a.^*

Entry	Substrate, R = CH_3_	Oxidant (Equiv)	Solvent, (Conc), and Additive (Equiv)	T (°C), Atmosphere, and Time	Product, R	Yield (%)	Ref
1	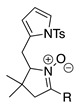	SeO_2_ (1.5)	dioxane (0.08 M)	rt argon 2.5 h	–CHO	79	[42]
2	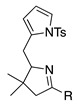	SeO_2_	– *^b^*	– *^b^*	–CHO	0	[42]
3	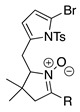	SeO_2_ (1.3)	dioxane (0.10 M)	rt argon 2.5 h	–CHO	43	[42]
4a	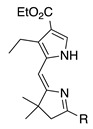	SeO_2_ (3.0)	dioxane (0.05 M)	rt argon 100 min	–CHO	40	[61]
4b	Same as 4a	SeO_2_ (3.0)	dioxane (0.05 M)	rt argon 1.5 h	–CHO	32	[22]
5	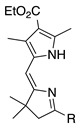	SeO_2_ (3.0)	dioxane (0.04 M)	rt argon 15 min	–CHO	66	[62]
6	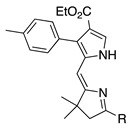	SeO_2_ (3.0)	dioxane (0.05 M)	rt argon 1 h	–CHO	39	[62]
7	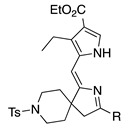	SeO_2_ (3.0)	dioxane (0.05 M)	rt argon 2 h	–CH(OMe)_2_	42	[61]
8	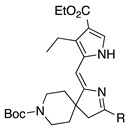	SeO_2_ (3.0)	dioxane (0.05 M)	rt argon 1 h	–CH(OMe)_2_	44	[61]
9	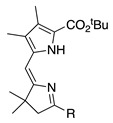	SeO_2_ (3.0)	dioxane (0.05 M)	rt argon 1.5 h	–CHO	63	[19]
10	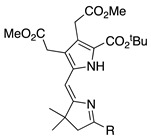	SeO_2_ (3.0)	dioxane (0.05 M)	rt – *^b^* 2 h	BC *^c^*	6.6	[19]
11	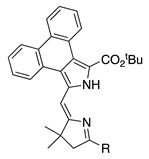	SeO_2_ (3.0)	dioxane (0.05 M)	rt argon 1.5 h	–CHO	55	[26]
12	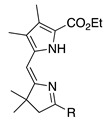	SeO_2_ (3.0)	dioxane (0.04 M)	rt argon 30 min	–CHO	59	[62]
13	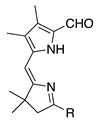	SeO_2_ (1.5)	dioxane (0.12 M)	rt argon 2 h	–CHO	99 *^d^*	[16]
14	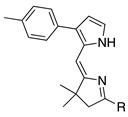	SeO_2_ (1.5)	dioxane (0.05 M)	rt argon 1.5 h	–CHO	47	[19]
15	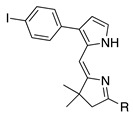	SeO_2_ (1.46)	dioxane (0.05 M)	rt – *^b^* 2 h	–CHO	57	[19]
16a	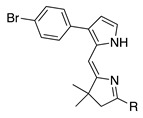	SeO_2_ (3.0)	dioxane (0.05 M)	rt argon 1.5 h	–CHO	22	[22]
16b *^e,f^*	Same as 16a	SeO_2_ (1.5)	dioxane (0.05 M)	rt air 0.5 h	–CHO	36 *^g^*	this work
16c *^e,f^*	Same as 16a	SeO_2_ (1.5)	dioxane (0.05 M) SiO_2_ (5.0 eq)	rt air 0.5 h	–CHO	38 *^g^*	this work
16d *^e,f^*	Same as 16a	SeO_2_ (1.5)	dioxane (0.05 M) pyridine (0.02 eq)	rt air 0.5 h	–CHO	28 *^g^*	this work
16e *^e,f^*	Same as 16a	SeO_2_ (1.5)	dioxane (0.05 M) C_6_F_5_CHO (1.5)	rt air 0.5 h	–CHO	18 *^g^*	this work
17 *^h^*	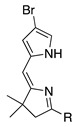	SeO_2_ (1.0–3.0)	dioxane (0.05 M)	0 °C to rt air or argon 15 min to 3 h	–CHO	0	this work
18	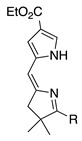	SeO_2_ (3.0)	dioxane (0.06 M)	rt – *^b^* 30 min	–CH(OMe)_2_	30	[20]
19	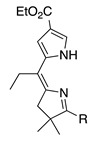	SeO_2_ (3.0)	dioxane (0.08 M)	rt – *^b^* 30 min	–CH(OMe)_2_	63	[20]
20	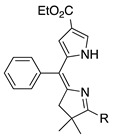	SeO_2_ (3.0)	dioxane (0.05 M)	rt – *^b^* 2 h	–CHO	65	[63]
21	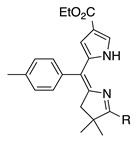	SeO_2_ (3.0)	dioxane (0.05 M)	rt – *^b^* 30 min	–CH(OMe)_2_	43	[20]
22	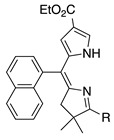	SeO_2_ (3.0)	dioxane (0.07 M)	rt – *^b^* 30 min	–CH(OMe)_2_	76	[20]
23	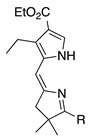	SeO_2_ (3.0)	dioxane (0.06 M)	rt – *^b^* 30 min	–CH(OMe)_2_	25	[20]
24	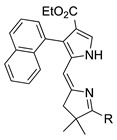	SeO_2_ (3.0)	dioxane (0.06 M)	rt – *^b^* 30 min	–CH(OMe)_2_	42	[26]
25	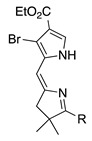	SeO_2_ (3.0)	dioxane (0.05 M) pyridine (0.02 eq)	rt – *^b^* 5 h	–CH(OMe)_2_	37	[26]
26	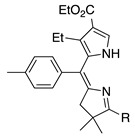	SeO_2_ (3.0)	dioxane (0.02 M)	rt – *^b^* 30 min	–CH(OMe)_2_	31	[20]
27	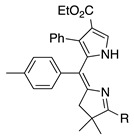	SeO_2_ (3.0)	dioxane (0.02 M)	rt – *^b^* 30 min	–CH(OMe)_2_	31	[20]
28	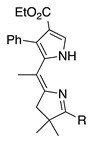	SeO_2_ (2.9)	dioxane (0.01 M)	rt – *^b^* 30 min	–CH(OMe)_2_	48	[20]
29	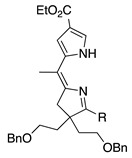	SeO_2_ (3.0)	dioxane (0.01 M)	rt – *^b^* 30 min	–CHO	57	[64]
30	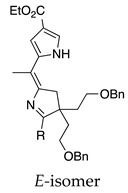	SeO_2_ (3.0)	dioxane (0.01 M)	rt – *^b^* 2 h	–CH(OMe)_2_	12, *E* 51, *Z*	[64]
31	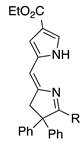	SeO_2_ (3.0)	dioxane (0.02 M)	rt – *^b^* 6 h	–CH(OMe)_2_	30, *Z* 25, *E*	[64]
32	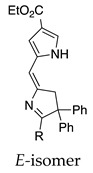	SeO_2_ (3.0)	dioxane (0.01 M)	rt – *^b^* 2 h	–CH(OMe)_2_	45, *E* 15, *Z*	[64]
33	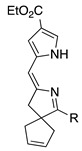	SeO_2_	– *^b^*	– *^b^*	–CHO	0	[64]
34	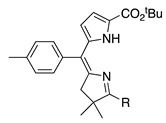	SeO_2_ (3.0)	dioxane (0.04 M)	rt – *^b^* 30 min	–CH(OMe)_2_	47	[20]
35	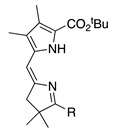	SeO_2_ (1.3)	DMF (0.18 M) pyridine (1.2 eq)	rt, then 80 – *^b^* 5 h and 15 min	–CHO	71	[18]
36a	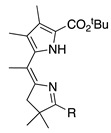	SeO_2_ (1.2)	DMF (0.11 M) pyridine (1.2 eq)	rt, then 80 – *^b^* 5 h and 15 min	–CHO	65	[18]
36b	Same as 36a	SeO_2_ (1.5)	dioxane (0.04 M)	reflux argon 30 min	–CHO	32	[16]
37	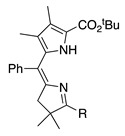	SeO_2_	DMF (0.09 M) pyridine (1.2 eq)	rt, then 80 – *^b^* 5 h and 15 min	–CHO	81	[18]
38	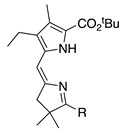	SeO_2_ (1.2)	DMF (0.11 M)	rt, then 80 – *^b^* 5 h and 15 min	–CHO	46	[65]
39a	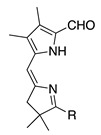	SeO_2_ (1.6)	dioxane (0.08 M)	rt argon 2 h	–CHO	68 *^d^*	[16]
39b	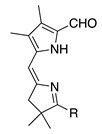	SeO_2_ (1.3)	CH_2_Cl_2_ (0.05 M) pyridine (1.3 equiv), then DMF (0.10) M	rt, then 80 – *^b^* 2 h and 15 min	–CHO	61	[17]
40a	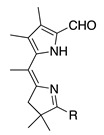	SeO_2_ (1.3)	dioxane (0.09 M)	rt argon 2 h	–CHO	99 *E*/*Z* 1:5 *^i^*	[16]
40b	Same as 40a	SeO_2_ (1.2)	CH_2_Cl_2_ (0.05 M) pyridine (1.19 equiv), then DMF (0.10) M	rt, then 80 – *^b^* 2 h and 15 min	–CHO	71	[17]
41a	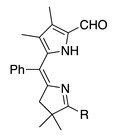	SeO_2_ (1.6)	dioxane (0.12 M)	rt argon 2 h	–CHO	62 *^d^*	[16]
41b	Same as 41a	SeO_2_ (1.0)	CH_2_Cl_2_ (0.05 M) pyridine (1.0 eq), then DMF (0.07 M)	rt, then 80 – *^b^* 5 h and 15 min	–CHO	70	[17]
42	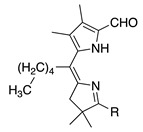	SeO_2_ (1.2)	CH_2_Cl_2_ (0.05 M) pyridine (1.2 eq), then DMF	rt, then 80 – *^b^* 5 h and 15 min	–CHO	63	[17]
43	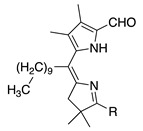	SeO_2_ (1.2)	CH_2_Cl_2_ (0.05 M) pyridine (1.2 eq), then DMF	rt, then 80 – *^b^* 5 h and 15 min	–CHO	65	[17]
44	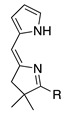	SeO_2_ (2.1)	CH_2_Cl_2_ (0.02 M)	rt argon – *^b^*	BC *^c^*	5.8	[20]
45	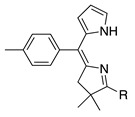	SeO_2_	– *^b^*	– *^b^*	–CHO	0	[20]

*^a^* Terms: dioxane refers to 1,4-dioxane (bp = 101 °C); rt = room temperature; DMF = *N*,*N*-dimethylformamide. *^b^* The reaction conditions including atmosphere or reaction time were not reported. *^c^* The product was converted to the bacteriochlorin (denoted BC); the yield is given for the overall transformation yielding the bacteriochlorin. *^d^* Reported to be unstable. *^e^* Each reaction used 0.02 mmol of 1-methyldihydrodipyrrin. *^f^* 1,3,5-Trimethoxybenzene (0.02 mmol) was used as an internal standard. *^g^* The yield was based on the –CHO proton versus the nine protons of the methyl groups of the internal standard using ^1^H-NMR spectroscopy. *^h^* Reaction with 1–3 equivalents of SeO_2_, a shorter reaction time, lower concentration, and dioxane (American Chemical Society grade) gave multiple spots upon thin layer chromatographic analysis, and no product was isolated. *^i^* Inseparable mixture.

**Scheme A1:**
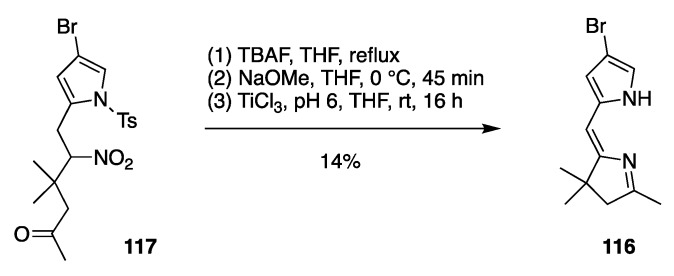
McMurry-type ring closure for dihydrodipyrrin formation.

## Abbreviations

2,6-DTBP	2,6-di-*tert*-butylpyridine
CVMW	closed-vessel microwave
DMF	*N,N*-dimethylformamide
ESI-MS	electrospray ionization mass spectrometry
MW	microwave
rt	room temperature
S_E_Ar	electrophilic aromatic substitution
TBAF	tetra-*n*-butylammonium fluoride
TBHP	*tert*-butyl hydroperoxide
TFA	trifluoroacetic acid
THF	tetrahydrofuran
TMSOTf	trimethylsilyl trifluoromethanesulfonate
TsOH·H_2_O	*p*-toluenesulfonic acid monohydrate
UHP	urea-hydrogen peroxide

## Appendix A

### Preparation of 8-Bromo-1,3,3-trimethyl-2,3-dihydrodipyrrin (116).

Following an established procedure [22], a sample of 4-bromo-2-(3,3-dimethyl-2-nitro-5-oxo-hexyl)-1-tosylpyrrole [67] (**117**, 3.43 g, 7.30 mmol) was treated with tetra-*n*-butylammonium fluoride (TBAF) (9.0 mL of 1.0 M in tetrahydrofuran (THF), 9.0 mmol; the THF was freshly distilled from Na/benzophenone ketyl) under an argon atmosphere and stirred for 1.5 h under reflux in an oil bath (Scheme A1). The reaction mixture was treated with saturated aqueous NaHCO_3_ followed by ethyl acetate. The organic layer was separated, dried (Na_2_SO_4_), and concentrated to a brown oil. The oil was further dried under high vacuum and purified by chromatography (silica, hexanes/ethyl acetate (3:2)) to afford the deprotected pyrrole as a yellow oil (501 mg); this product was used directly in the next step. For the ensuing TiCl_3_-catalyzed reductive cyclization, a solution of the pyrrole (501 mg, 1.58 mmol) in THF (10 mL; freshly distilled from Na/benzophenone ketyl) was deaerated by bubbling with argon for 10 min, and then treated with NaOMe (430 mg, 7.90 mmol) followed by stirring for 45 min at 0 °C under argon. In the second flask, a solution of NH_4_OAc (48.7 g, 632 mmol) in water (63 mL) was deaerated by bubbling with argon for 30 min and then treated with TiCl_3_ (1.22 g, 7.90 mmol, 20% *w/v* solution in 2 N HCl). The suspension was stirred for 15 min at room temperature under an atmosphere of argon. The solution in the first flask containing the nitronate anion of the free pyrrole was transferred via a cannula to the buffered TiCl_3_ solution in the second flask. The resulting brown mixture was stirred for 1 h under argon, and the flask was sealed to react for 16 h. The reaction mixture was slowly poured into a stirred mixture of saturated aqueous NaHCO_3_ (350 mL) and ethyl acetate (200 mL). The entire mixture was stirred vigorously at room temperature for 15 min. A clear phase separation did not occur. An additional quantity (200 mL) of ethyl acetate was added. The organic layer was separated and washed with saturated aqueous NaHCO_3_ (200 mL × 3). The dark-orange organic layer was dried (Na_2_SO_4_) and concentrated to a dark oil. The resulting oil was passed through a silica column (silica, hexanes/ethyl acetate (3:1)) to afford the title compound as a brown oil (83 mg, 14%): ^1^H-NMR (300 MHz, rt, CDCl3): δ 1.18 (s, 6 H), 2.20 (s, 3 H), 2.50 (s, 2 H), 5.62 (s, 1 H), 6.06–6.08 (m, 1 H), 6.76–6.77 (m, 1 H), 10.8 (br s, 1 H); ^13^C{^1^H}-NMR (75 MHz, rt, CDCl_3_) *δ* 20.8, 29.2, 41.2, 53.8, 96.4, 103.4, 109.9, 118.1, 131.9, 177.7. Electrospray ionization mass spectrometry (ESI-MS) analysis gave the characteristic double peaks owing to the isotopes of bromine (^79^Br, ^81^Br) at *m/z* ~267 and ~269 Da; here, data are listed for both ions: obsd 267.0498, calcd 267.0491 [(M + H)^+^, M = C_12_H_15_N_2_^79^Br]; obsd 269.0476, calcd 269.0471 [(M + H)^+^, M = C_12_H_15_N_2_^81^Br]. Studies of this compound are described in entry 17 of Table 1.

## References

[1] Riley H.L., Morley J.F., Friend N.A.C. (1932). Selenium Dioxide, a New Oxidising Agent. Part I. Its Reaction with Aldehydes and Ketones. J. Chem. Soc..

[2] Riley H.L., Friend N.A.C. (1932). Selenium Dioxide, a New Oxidising Agent. Part II. Its Reaction with Some Unsaturated Hydrocarbons. J. Chem. Soc..

[3] Astin S., Newman A.C.C., Riley H.L. (1933). Selenium Dioxide, a New Oxidising Agent. Part III. Its Reaction with Some Alcohols and Esters. J. Chem. Soc..

[4] Emeléus H.J., Riley H.L. (1933). The Luminous Reduction of Selenium Dioxide. Proc. Roy. Soc. Lond. Ser. A.

[5] Astin S., Riley H.L. (1934). Selenium Dioxide. A New Oxidising Agent. Part IV. The Preparation and Properties of Ethyl Ketohydroxysuccinate. J. Chem. Soc..

[6] Astin S., Moulds L.D.V., Riley H.L. (1935). Selenium Dioxide, a New Oxidising Agent. Part V. Some Further Oxidations. J. Chem. Soc..

[7] Moulds L.D.V., Riley H.L. (1938). The Polymerisation of Methylglyoxal. J. Chem. Soc..

[8] Riley H.L. (1947). Oxidation Activity of Selenium Dioxide. Nature.

[9] Blayden H.E. (1987). In Memoriam: HL Riley. Carbon.

[10] Rabjohn N. (1948). Selenium Dioxide Oxidation. Org. React..

[11] Waitkins G.R., Clark C.W. (1945). Selenium Dioxide: Preparation, Properties, and Use as Oxidizing Agent. Chem. Rev..

[12] Nakamura A., Nakada M. (2013). Allylic Oxidations in Natural Product Synthesis. Synthesis.

[13] Mlochowski J., Wojtowicz-Mlochowska H. (2015). Developments in Synthetic Application of Selenium(IV) Oxide and Organoselenium Compounds as Oxygen Donors and Oxygen-Transfer Agents. Molecules.

[14] Scheer H., Grimm B., Porra R.J., Rüdiger W., Scheer H. (2006). An Overview of Chlorophylls and Bacteriochlorophylls: Biochemistry, Biophysics, Functions and Applications. Chlorophylls and Bacteriochlorophylls. Biochemistry, Biophysics, Functions and Applications.

[15] Lindsey J.S. (2015). *De Novo* Synthesis of Gem-Dialkyl Chlorophyll Analogues for Probing and Emulating our Green World. Chem. Rev..

[16] Jacobi P.A., Lanz S., Ghosh I., Leung S.H., Löwer F., Pippin D. (2001). A New Synthesis of Chlorins. Org. Lett..

[17] O’Neal W.G., Roberts W.P., Ghosh I., Wang H., Jacobi P.A. (2006). Studies in Chlorin Chemistry. 3. A Practical Synthesis of C,D-Ring Symmetric Chlorins of Potential Utility in Photodynamic Therapy. J. Org. Chem..

[18] O’Neal W.G., Jacobi P.A. (2008). Toward a General Synthesis of Chlorins. J. Am. Chem. Soc..

[19] Zhang S., Kim H.-J., Tang Q., Yang E., Bocian D.F., Holten D., Lindsey J.S. (2016). Synthesis and Photophysical Characteristics of 2,3,12,13-Tetraalkylbacteriochlorins. New J. Chem..

[20] Liu Y., Lindsey J.S. (2016). Northern–Southern Route to Synthetic Bacteriochlorins. J. Org. Chem..

[21] Wang P., Lu F., Lindsey J.S. (2020). Use of the Nascent Isocyclic Ring to Anchor Assembly of the Full Skeleton of Model Chlorophylls. J. Org. Chem..

[22] Zhang S., Lindsey J.S. (2017). Construction of the Bacteriochlorin Macrocycle with Concomitant Nazarov Cyclization to Form the Annulated Isocyclic Ring: Analogues of Bacteriochlorophyll *a*. J. Org. Chem..

[23] Ptaszek M., Bhaumik J., Kim H.-J., Taniguchi M., Lindsey J.S. (2005). Refined Synthesis of 2,3,4,5-Tetrahydro-1,3,3-trimethyldipyrrin, a Deceptively Simple Precursor to Hydroporphyrins. Org. Process Res. Dev..

[24] Ninomiya I., Hashimoto C., Kiguchi T., Naito T., Barton D.H.R., Lusinchi X., Milliet P. (1990). Dehydrogenation with Benzeneseleninic Anhydride in the Total Synthesis of Ergot Alkaloids. J. Chem. Soc. Perkin Trans 1.

[25] Trachtenberg E.N., Augustine R.L. (1969). Selenium Dioxide Oxidation. Oxidation: Techniques and Applications in Organic Synthesis.

[26] Fujita H., Jing H., Krayer M., Allu S., Veeraraghavaiah G., Wu Z., Jiang J., Diers J.R., Magdaong N.C.M., Mandal A.K. (2019). Annulated Bacteriochlorins for Near-Infrared Photophysical Studies. New J. Chem..

[27] Clark K.J., Fray G.I., Jaeger R.H., Robinson R. (1959). Synthesis of D- and L- *Iso*iridomyrmecin and Related Compounds. Tetrahedron.

[28] Bhalerao U.T., Rapoport H. (1971). Stereochemistry of Allylic Oxidation with Selenium Dioxide. Stereospecific Oxidation of *gem*-Dimethyl Olefins. J. Am. Chem. Soc..

[29] Camps F., Coll J., Parente A. (1978). Selenium Dioxide Oxidation of Substrates with Acid Labile Groups. Synthesis.

[30] Umbreit M.A., Sharpless K.B. (1977). Allylic Oxidation of Olefins by Catalytic and Stoichiometric Selenium Dioxide with *tert*-Butyl Hydroperoxide. J. Am. Chem. Soc..

[31] Kalsi P.S., Chhabra B.R., Singh J., Vig R. (1992). Selective Oxidation of Primary Allylic Alcohols to α,β-Unsaturated Aldehydes. Synlett.

[32] Kuwajima I., Shimizu M., Urabe H. (1982). Oxidation of Alcohols with *tert*-Butyl Hydroperoxide and Diaryl Diselenide. J. Org. Chem..

[33] van der Toorn J.C., Kemperman G., Sheldon R.A., Arends I.W.C.E. (2009). Diphenyldiselenide-Catalyzed Selective Oxidation of Activated Alcohols with *tert*-Butyl Hydroperoxide: New Mechanistic Insights. J. Org. Chem..

[34] Nagaoka H., Shibuya K., Yamada Y. (1994). Total Synthesis of Upial, A Marine Sesquiterpene Possessing Bicyclo [3.3.1]nonane Ring System. Tetrahedron.

[35] Shibuya K. (1994). A Novel Allylic Oxidation Using a Combination of Formic Acid and Selenium Dioxide. Synth. Commun..

[36] Koltun E.S., Kass S.R. (2000). Synthesis of Cycloocta-3,5-dien-1-ol and Cycloocta-3,5-dien-1-one: SeO_2_/O_2_ Oxidation of Dienes. Synthesis.

[37] Hanold N., Meier H. (1985). Die Unsymmetrischen Cyclooctadienine: 1,3-Cyclooctadien-5-in und 1,6-Cyclooctadien-3-in. Chem. Ber..

[38] Javaid K.A., Sonoda N., Tsutsumi S. (1969). A New Reaction in the Selenium Dioxide Oxidation of Olefins. Tetrahedron Lett..

[39] Sonoda N., Yamamoto Y., Murai S., Tsutsumi S. (1972). A New Acid Catalyzed Oxidation of Some Acetylenic Compounds with Selenium Dioxide. Chem. Lett..

[40] Bender H., Döpp D. (1980). 2*H*-Pyrrole-l-oxides by Selenium Dioxide Dehydrogenation of Pyrroline-1-oxides. Tetrahedron Lett..

[41] Sakamoto T., Sakasai T., Yamanaka H. (1981). Studies on Pyrimidine-derivatives. XXII. Site-selective Oxidation of Dimethylpyrimidines with Selenium Dioxide to Pyrimidine-monoaldehydes. Chem. Pharm. Bull..

[42] Kim H.-J., Dogutan D.K., Ptaszek M., Lindsey J.S. (2007). Synthesis of Hydrodipyrrins Tailored for Reactivity at the 1- and 9-Positions. Tetrahedron.

[43] Back T.G., Kerr R.G. (1982). Oxidation of 1,l-Disubstituted Hydrazines with Benzeneseleninic Acid and Selenium Dioxide. Facile Preparation of Tetrazenes. Can. J. Chem..

[44] Remias J.E., Sen A. (2003). Nitrogen Oxides/Selenium Dioxide-Mediated Benzylic Oxidations. J. Mol. Catal. A Chem..

[45] Goswami S., Adak A.K. (2003). Microwave Assisted Improved Synthesis of 6-Formylpterin and Other Heterocyclic Mono- and Di-aldehydes. Synth. Commun..

[46] Belsey S., Danks T.N., Wagner G. (2006). Microwave-Assisted Selenium Dioxide Oxidation of Camphor Derivatives to α-Dicarbonyl Compounds and Oxoimines. Synth. Commun..

[47] Shirude S.T., Patel P., Giridhar R., Yadav M.R. (2006). An Efficient and Time Saving Microwave-Assisted Selenium Dioxide Oxidation of 1,2-Diarylethanones. Ind. J. Chem..

[48] Manktala R., Dhillon R.S., Chhabra B.R. (2006). Urea-Hydrogen Peroxide and Microwave: An Eco-friendly Blend for Allylic Oxidation of Alkenes with Catalytic Selenium Dioxide. Ind. J. Chem..

[49] Gelman D.M., Perlmutter P. (2009). Microwave-assisted Selenium Dioxide Mediated Selective Oxidation of 1-Tetralones to 1,2-Naphthoquinones. Tetrahedron Lett..

[50] Riley H.A., Gray A.R. (1935). Phenylglyoxal. Org. Syn..

[51] Riley H.A., Gray A.R. (1943). Phenylglyoxal. Org. Syn. Coll. Vol..

[52] Trachtenberg E.N., Nelson C.H., Carver J.R. (1970). Mechanism of Selenium Dioxide Oxidation of Olefins. J. Org. Chem..

[53] Arigoni D., Vasella A., Sharpless K.B., Jensen H.P. (1973). Selenium Dioxide Oxidations of Olefins. Trapping of the Allylic Seleninic Acid Intermediate as a Seleninolactone. J. Am. Chem. Soc..

[54] Sharpless K.B., Gordon K.M. (1976). Selenium Dioxide Oxidation of Ketones and Aldehydes. Evidence for the Intermediacy of β-Ketoseleninic Acids. J. Am. Chem. Soc..

[55] Stephenson L.M., Speth D.R. (1979). Mechanism of Allylic Hydroxylation by Selenium Dioxide. J. Org. Chem..

[56] Woggon W.-D., Ruther F., Egli H. (1980). The Mechanism of Allylic Oxidation by Selenium Dioxide. J. Chem. Soc. Chem. Commun..

[57] Warpehoski M.A., Chabaud B., Sharpless K.B. (1982). Selenium Dioxide Oxidation of Endocyclic Olefins. Evidence for a Dissociation-Recombination Pathway. J. Org. Chem..

[58] Singleton D.A., Hang C. (2000). Isotope Effects and the Mechanism of Allylic Hydroxylation of Alkenes with Selenium Dioxide. J. Org. Chem..

[59] Ra C.S., Park G. (2003). Ab Initio Studies of the Allylic Hydroxylation: DFT Calculation on the Reaction of 2-Methyl-2-butene with Selenium Dioxide. Tetrahedron Lett..

[60] Greenwood N.N., Earnshaw A. (2001). Chemistry of the Elements.

[61] Reddy K.R., Lubian E., Pavan M.P., Kim H.-J., Yang E., Holten D., Lindsey J.S. (2013). Synthetic Bacteriochlorins with Integral Spiro-piperidine Motifs. New J. Chem..

[62] Zhang S., Reddy M.N., Mass O., Kim H.-J., Hu G., Lindsey J.S. (2017). Synthesis of Tailored Hydrodipyrrins and Their Examination in Directed Routes to Bacteriochlorins and Tetradehydrocorrins. New J. Chem..

[63] Reddy M.N., Zhang S., Kim H.-J., Mass O., Taniguchi M., Lindsey J.S. (2017). Synthesis and Spectral Properties of *meso*-Arylbacteriochlorins Including Insights into Essential Motifs of Hydrodipyrrin Precursors. Molecules.

[64] Liu Y., Allu S., Reddy M.N., Hood D., Diers J.R., Bocian D.F., Holten D., Lindsey J.S. (2017). Synthesis and Photophysical Characterization of Bacteriochlorins Equipped with Integral Swallowtail Substituents. New J. Chem..

[65] Noboa M.A., AbuSalim D.I., Lash T.D. (2019). Azulichlorins and Benzocarbachlorins Derived Therefrom. J. Org. Chem..

[66] Artico M., Jones R.A. (1990). Nitration, Sulfonation, and Halogenation. Pyrroles. Part One. The Synthesis and the Physical and Chemical Aspects of the Pyrrole Ring.

[67] Krayer M., Balasubramanian T., Ruzié C., Ptaszek M., Cramer D.L., Taniguchi M., Lindsey J.S. (2009). Refined Syntheses of Hydrodipyrrin Precursors to Chlorin and Bacteriochlorin Building Blocks. J. Porphyr. Phthalocyanines.

